# Wanting to provide the childhood they never got – a meta-synthesis of how survivors of childhood abuse experience becoming a parent

**DOI:** 10.1080/17482631.2025.2523175

**Published:** 2025-06-23

**Authors:** Signe Hjelen Stige, Martin Mauritzson Greve, Kari Anne Trefall

**Affiliations:** Department of Clinical Psychology, University of Bergen, Bergen, Norway

**Keywords:** Abuse, trauma, childhood, parenthood, parent

## Abstract

**Purpose:**

Experiencing childhood abuse is associated with an increased risk of developing a broad range of health issues and might impact the experience of transitioning into parenthood. We therefore wanted to explore how survivors of childhood abuse experience becoming a parent.

**Methods:**

Using the method of meta-ethnography, we synthesized the findings from 13 primary studies.

**Results:**

The meta-synthesis resulted in three themes: 1) Own experiences of abuse enhance the desire to be a good parent; 2) Own experiences of abuse challenges parents in their new role; and 3) Becoming a parent as an opportunity to start a healing process.

**Conclusion:**

Our results indicated that parents shared a dedication to providing their children with a better childhood than they had experienced. However, many parents struggled with low self-efficacy, and several became aware of their difficulties with attachment and emotional regulation when they became parents. Although challenging, the parenting role was also a source of positive and healing experiences. The implications of these results are discussed.

## Introduction

Experiencing abuse like physical assault, emotional abuse, neglect, or sexual abuse from caregivers during childhood is unfortunately a relatively frequently occurring phenomena with great costs—both at an individual and societal level (Pereznieto et al., 2014). The World Health Organization (WHO) estimates that 75% of children in the age group 2–4 years worldwide experience physical or psychological abuse from their caregivers and 20% of girls and 7.5% of boys are exposed to child sexual abuse (World Health Organization, 2022). Research also shows that exposure to one type of abuse increases the risk of being exposed to other types of abuse (Finkelhor et al., 2009). Experiences of abuse have consistently been linked to an increased risk for a range of both somatic and mental health problems (Finkelhor et al., 2009), and it has been estimated that childhood abuse has a societal cost of staggering seven billion dollars, or 8% of the world’s BNP (Pereznieto et al., 2014).

The transition into parenthood is experienced as challenging by many parents, regardless of carrying experiences with abuse, due to substantial changes in their life situation with increased responsibilities and new tasks (Nyström & Öhrling, 2004). There are some indications, however, that parents with a history of childhood abuse might experience the transition into parenthood as particularly difficult. They might for example struggle with low self-efficacy and increased levels of stress due to aftereffects of their own experiences with abuse and lack good role models (e.g., Hughes & Cossar, 2016; Steele et al., 2016). In line with this, research has found that experiences with trauma can affect parenting (Iyengar et al., 2014; Schore, 2002), and lead to intergenerational trauma (Babcock Fenerci et al., 2016; Kellermann, 2001). This makes it particularly important to explore how parents with a history of childhood abuse experience the transition into parenthood.

In line with this, we have located two previous meta-syntheses during our systematic literature search that explore similar topics. Siverns and Morgan (2019) synthesized 11 studies using a trauma-theoretical lens to explore the parenting experience among survivors of childhood abuse. They reported that parents’ identity was formed through the lens of trauma, focused on protecting their children from harm, and needed support from others. Herbell and Bloom (2020) synthesized 11 studies, focusing on the parenting practices among mothers with adverse childhood experiences. They reported how mothers worked to break the cycle of abuse, described strategies to protect their children, and seek support. At the same time, they described a pervasive fear of authorities removing their child.

## Rationale and aims

Most existing studies on how childhood abuse affects parenting are quantitative (e.g., Greene et al., 2020; Iyengar et al., 2014). We wanted to expand on existing meta-synthesis by including newer qualitative studies, including experiences from both mothers and fathers, and openly explore parents’ experiences from a wider perspective than a strict trauma focus (i.e., Siverns & Morgan, 2019). This last point was important for us, as we regard childhood abuse as a significant and severe category of trauma exposure that is associated with increased risk of a range of health issues (e.g (Finkelhor et al., 2009), but we do not assume that exposure to abuse automatically results in trauma-specific symptoms. By exploring experiences of parenthood in the context of childhood abuse, specifically experiences with neglect, physical violence, psychological abuse, sexual abuse or witnessing domestic violence, we hoped to offer new insights to researchers, parents, and health professionals supporting them. In this study, we therefore compiled existing qualitative studies in the field, to synthesize parents’ experiences of the transition into parenthood in the context of childhood abuse across studies and contexts.

## Methods

To address our study aim, we chose to use the method of qualitative meta-synthesis, which allows comparison and integration of findings across existing qualitative studies to provide new knowledge about a phenomenon (Britten et al., 2002; Sandelowski, 2012). In the analysis and synthesis of the included studies, we relied on the meta-ethnographic comparative method (Noblit & Hare, 1999; Sattar et al., 2021) in combination with more systematic coding inspired by reflexive thematic analysis (Braun & Clarke, 2019).

### Search strategy and selection of studies

To identify relevant primary studies to include in the meta-synthesis (step 2 in meta-ethnography; Noblit & Hare, 1999) we articulated a search strategy in collaboration with a university librarian. We decided to only include peer-reviewed articles using qualitative and mixed-methods designs. To make the identification of relevant articles feasible, the search was limited to articles published within the last 20 years. For details on search terms and inclusion criteria, see Table 1.

**Table 1.** Search strategy and inclusion criteria.

Search terms and strategy	Inclusions criteria
(child* NEAR/2 (violen* OR abus* OR trauma* OR advers* OR neglect*) OR “trauma assessment” OR “early life harshness” OR violen* NEAR/2 child*) AND (becom* NEAR/2 (mother* OR father* OR parent* OR caregiver) OR motherhood OR fatherhood OR parenthood OR parent* NEAR/2 (style OR behav*) OR postnatal) AND (impact OR interview* OR experienc* OR qualitativ* OR narrative OR phenomenolog* OR interpret* OR identify* OR address* OR inquir* OR expos* OR “grounded theory” OR analysis)	1. Participants exposed to abuse (physical, psychological, sexual) and/or neglect by care giver before age of 16 2. Articles contain direct quotes from qualitative studies 3. Participants have been interviewed about their experiences as parents/care givers for children 4. Articles are written in English or Norwegian 5. Articles have been peer reviewed and are published within the last 20 years

**Figure 1.**
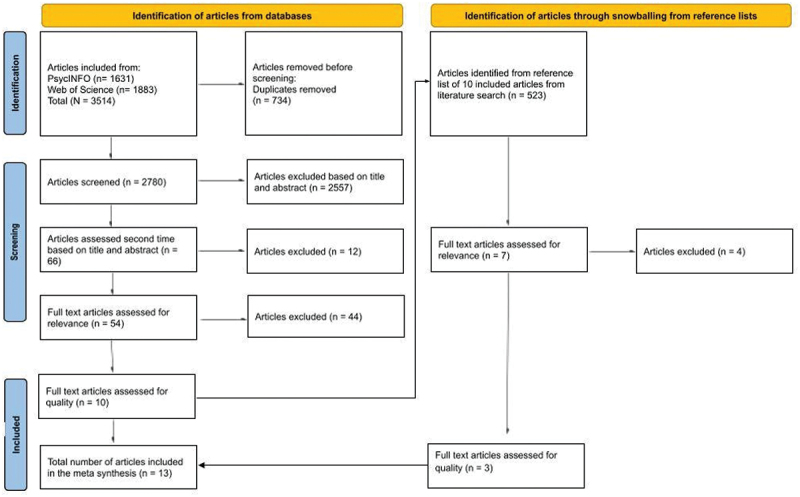
Flow diagram selection process.

The search was conducted in PsychINFO and Web of Science (WOS) in April 2023 and resulted in 1631 references from PsychINFO and 1883 references from WOS, that were imported in EndNote (The EndNote Team, 2013). Duplicates were removed first through the function in EndNote (removing 501 duplicates) before the second and third author manually went through all references removing 233 additional duplicates. The literature search thus resulted in 2780 unique articles. Assessment of fit and inclusion of articles was done by the second and third author under supervision of the first author. First, the second and third author assessed the first 10 articles in collaboration to establish a common understanding of the inclusion criteria. The next 90 titles and abstracts were assessed individually by both the second and third author, to establish consensus. Responsibility for assessment of inclusion for the remaining 2680 articles was divided between the second and third author. This process resulted in the selection of 54 relevant articles, that were read in full to assess fit with inclusion criteria. This resulted in the exclusion of 44 articles, leaving 10 included studies from the systematic search. Because there is a lack of standardized search terms for qualitative research methods, we assessed the reference lists of the 10 included articles to identify potential articles filling our inclusion criteria that we had not located through our systematic search. Seven of the 523 references were a potential fit, and these articles were assessed for inclusion, resulting in three additional articles being included. See Figure 1 for an overview of the process of identifying and selecting included studies.

### Quality assessment and included studies

The 13 included studies were then assessed for quality using the Critical Appraisal Skills Programme (CASP, 2018) in combination with the “Critique” items from the EPICURE evaluation agenda (B. Stige et al., 2009). None of the 13 articles were excluded based on quality, resulting in a total of 13 articles being included in the meta-synthesis.

The included articles were published between 2009 and 2022 and were conducted in the US (*n* = 5), UK (*n* = 2), Ireland (*n* = 1), Canada (*n* = 1), Australia (*n* = 1), Israel (*n* = 1), South Africa (*n* = 1), and Sweden (*n* = 1). All studies were conducted within one specific cultural context, and none of the studies explored cultural aspects of the phenomena in their analysis. Most studies relied on small samples (3–19 participants), except for three studies from the US exploring the experience of motherhood and support needs among survivors of CSA. Most studies only included participants from one gender (9 women only, 2 men only). Only two out of the 13 included studies included participants from more than one gender. A total number of 197 persons (173 women) aged 20–72 had participated in the 13 studies. Although all studies fulfilled our inclusion criteria, there was a great variation in the focus for the 13 studies. Some studies narrowed the focus to a particular type of abuse (in all cases CSA). Seven studies hence focused on mothers and one on fathers with experiences of CSA. Within this group of studies some adopted a broader focus (e.g., experience of parenthood), while others adopted a narrower focus (e.g., experience with breastfeeding or intergenerational trauma). Other studies focused more on challenging developmental contexts. Two studies hence focused on parents with experiences from the foster care system, one study focused mothers who grew up with a caretaker with psychosis, and one study on parents who grew up with a caretaker who abused substances. There was therefore a great heterogeneity in focus, contexts, and participants across the included studies. For more information about the included studies, see Table 2.

**Table 2.** Overview and description of the 13 included articles.

First author and year	Purpose of study	Recruitment strategy	Participants	Country	Qualitative methods used	Primary findings/themes	Abuse exposure
Aparicio et al. (2015)	Explore the experience of motherhood among teenage mothers in foster care with a history of abuse.	Not provided	6 teenage mothers in foster care with a history of abuse	US	Semi structured interviews. Each participant was interviewed three times. Interpretative phenomenological analysis (IPA)	1. Darkness and despair 2. Glimpses of light in the darkness 3. New Beginnings	N*: 6 PV*: 4 SA*: 2 WDV*: 4
Byrne et al. (2017)	Explore consequences of experiences with CSA on women’s experience of pregnancy, birth, and postnatal period.	Participants recruited through local psychological healthcare. Clinicians contacted potential participants.	3 mothers (27–40 years) with experiences with CSA	UK	Semi structured interviews. Each participant was interviewed three times. Narrative analysis	1. Experience of identity 2. Experience of embodiment 3. Experience of parenting	SA*: 3
Cavanaugh et al. (2015)	Explore the needs and experiences of mothers with a history of CSA.	Participants recruited through advertisement.	44 mothers (20–58 years) with experiences with CSA	US	Semi structured interviews. Thematic analysis	1. Being a parent 2. Hopes and desires for the future	SA*:44 Unknown number: PV*,WDV*
Coles (2009)	Explore survivors of CSA’s experience with breast feeding.	Participants recruited through advertisement.	11 mothers at different stages in the process of breast feeding (from 6 weeks to 2 years after giving birth)	Australia	Semi structured interviews. Five of the mothers were interviewed twice. Thematic analysis	1. Enhancement of the mother—baby relationship by breastfeeding 2. Validation of the maternal body through breastfeeding 3. Coping with breastfeeding through a maternal—sexual split4. Breastfeeding in public raising issues of exposure and control	SA*: 11
Dandy et al. (2020)	Explore the experience of being a father among people with experience from foster care who now care for their own children.	Participants recruited through advertisement at an NGO supporting persons with experience from foster care.	5 fathers (25–66 years) who have grown up in foster care	UK	Semi structured interviews. Interpretative phenomenological analysis (IPA)	1. Going back to move forward 2. Reliving the past 3. Breaking the cycle	Unknown type ACE: «maltreatment and abuse»
Gichaz et al. (2022)	Explore parenting in an intergenerational perspective among survivors of CSA.	Participants recruited through information leaflets at crisis centre for sexual abuse.	19 mothers (60–72 years)	Israel	Semi structured interviews. Interpretative phenomenological analysis (IPA)	1. «How did I raise those kids? I really do not know»: Early parenthood experiences of ageing women incest survivors 2. «Is it possible that something good came out of it? » Adult children as a victory over incest experiences	SA*: 19 Unknown number: PV
Haiyasoso and Trepal (2019)	Explore how survivors of CSA experience parenthood.	Participants recruited through advertisement and emails to local universities.	9 mothers (24–61 years)	US	Semi structured interviews. Narrative analysis	1. Negotiating a balance of protecting and letting go 2. Using relational images as guideposts for parenting decisions 3. Exploring functioning in relational contexts	SA*: 9 Not provided information on other types of ACE
Kadish (2015)	Explore how women experience to be influenced by their experiences of growing up with a caretaker with psychosis.	Convenience sampling. Participants recruited through their knowledge of the research group.	5 mothers (28–53 years)	South- Africa	Semi structured interviews. Thematic content analysis with interpretivist-constructivist approach.	1. Maternal psychosis, motherhood, and feminine identity	Unknown type ACE
Lange et al. (2020)	Explore how survivors of CSA experience parenting and need for support.	Participants recruited through contact with different NGOs working with abuse, violence, mental health and parenting.	35 mothers (20–40 years) with 1–5 children	US	Digital questionnaire: Childhood Trauma Questionnaire Short Form (CTQ-SF), The Center for Epidemiological Studies Depression Scale (CES-D); Semi structured interviews. Thematic analysis	1. Protection from abuse 2. Abuse of child 3. Mother-child relationship 4. Breastfeeding 5. Perception of child and motherhood 6. Coping	SA*: 35 PA*: 13 PV*: 14 Emotional N*:14 Physical N*: 10
Matthews and Desjardins (2019)	Explore how adults with experiences of childhood abuse picture a family.	Participants recruited through advertisement.	15 participants (13 women), mean age 30 years	Canada	Two interviews with each participant: one life story interview and one semi structured interview. Thematic analysis	1. Meant to be mothers: Filtering and transforming dysfunction	PA*: 15 PV*: 12 N*: 11 WDV: 6
O’Brien et al. (2019)	Explore survivors of CSA’s experience of being a father.	Participants recruited through three NGOs for adult survivors of CSA.	11 fathers (mean age 45 years) with 1–4 children	Irland	Semi structured in-depth interviews. Interpretative Phenomenological approach (IPA)	1. Confronting the tormented self in fatherhood: «It opened up a wound that was already there» 2. Restoring the Self through Fatherhood: «When you hold your children in your arms that helps you to change»	SA*: 11 Not provided information on other types of ACE
Tedgard et al. (2018)	Explore how growing up with caretakers abusing substances is experienced to influence people as parents.	Participants recruited through intervention programs for newborns mental health.	19 parents (13 mothers), no couples. Children aged 1–5 years.	Sweden	Semi structured interview. Qualitative content analysis.	1. Inadequate support for the development of functional affect regulation 2. Challenges in being a parent oneself	Emotional N*:18 PA: 15 PV*: >8 WDV*: ≥ 1
Wright et al. (2012)	Explore survivors of CSA’s experience of being a mother.	Participants recruited through advertisement.	79 mothers (mean age 38.2 years), children’s age varied from a few months to adulthood.	US	Written narratives from all participants. Semi structured interviews with 15 participants. Grounded Theory.	1. The hard work of mothering as a survivor 2. Developing a mothering self	SA*: 79 Not provided information on other types of ACE

N* = Neglect, PV*= Physical violence, PA*= Psychological abuse, SA*= Sexual abuse, WDV*: Witnessing domestic violence.

### The process of synthesizing findings across studies

Qualitative meta-synthesis allows comparison and integration of findings across existing qualitative studies, even when there is heterogeneity in the primary studies, as described above. Since we relied on the meta-ethnographic comparative method, our focus was on comparison and translation of findings across studies. The process of synthesizing findings from the 13 included studies therefore results in findings that integrate data from all included studies. The synthesis is, however, limited to the information included in the primary studies. There might therefore be several aspects that are relevant to the research question that a qualitative meta-synthesis will not be able to address due to research gaps and understudied groups and phenomena.

The qualitative meta-synthesis was done by the second and third author under supervision of the first author. The process of synthesizing findings across the primary studies started with summarizing and comparing the findings in the 13 included studies (step 3 and 4 in meta-ethnography; Noblit & Hare, 1999), using the platform Miro (2023) as a visual support. In step 5 and 6 in meta-ethnography (Noblit & Hare, 1999; Sattar et al., 2021) the focus is on translating the study findings into each other and synthesizing these translations to articulate new knowledge based on the included studies. To do this, we alternated between line-by-line coding of the result sections of the 13 included studies, inspired by reflexive thematic analysis (Braun & Clarke, 2019) and the use of Miro to look at the more overarching meaning patterns. NVivo 12 (Lumivero, 2017) was used as technical support for the line-by-line coding. The final result of the synthesis and the presentation of this in a thematic structure happened through a series of meetings between all authors where analysis and synthesis were refined, and with the final adjustments happening as we were writing up the findings (step 7 of meta-ethnography; Noblit & Hare, 1999). All 13 studies contributed to all the themes in the presented thematic structure.

### Researchers and reflexivity

The first author is a mother and a professor in clinical psychology who has published substantially within the field of psychological trauma and is very experienced in the use of qualitative research methods, including qualitative meta-synthesis. She was the supervisor of the second and third authors when they were writing their Master thesis. This article is a revised version of the Master thesis. The second author is a newly educated clinical psychologist, a Norwegian man in his early thirties, not a father, not published, but with an interest with the field of psychological trauma. The third author is a newly educated clinical psychologist. She is not yet a parent but has had an interest in subjects revolving around developmental psychology, families, parenting and psychological trauma. Throughout the process the research team has used their converging and diverging position to support reflexive processes, including the second and third authors’ positions for interpreting experiences of a phenomenon of which they have no personal experience (parenthood).

### Ethics approval statement

No ethical approval was needed for this study as we only included articles that have already been published as our data material.

## Results

The analysis and synthesis of the result sections in the 13 included studies resulted in the construction of three main themes; *1) Own experiences of abuse enhance the desire to be a good parent; 2) Own experiences of abuse challenges parents in their new role;* and *3) Becoming a parent as an opportunity to start a healing process.*

### Theme 1: Own experiences of abuse enhance the desire to be a good parent

All parents across all studies talked about a difficult childhood that had influenced them in different ways. Across studies participants shared experiences of their own caretakers not being there for them the way they had needed. This elicited a strong motivation in many participants to prevent their own children having similar experiences. At the same time, many participants felt scarred by their own experiences and had seen abuse being passed down generations. This elicited a strong fear that they would not succeed in their endeavour to be good enough parents to their own children.

In most studies several participants shared their wish for their own children to have a better childhood than what they themselves had lived through. They wished to use their experiences of abuse to be a different type of parent than their own parents had been: “I really dislike the way my parents were, and I don’t want to be like my mother at all” (Tedgard et al., 2018, p. 6). Participants in most studies reflected upon how they wanted to provide a safe and stable home, where they were available to their children: “I just wanted a stable place for him” (Aparicio et al., 2015, p. 48). This became the most important thing for the participants: “I think other people … want their kids to be doctors or brain surgeons or x, y, z. I just wanted my kids to get through childhood unscathed” (Haiyasoso & Trepal, 2019, p. 287).

While participants across most studies expressed a very strong motivation to be a good parent, many participants also experienced a strong fear of falling short and felt that their background meant they had to prove to others, and themselves, that they were good enough parents: “It triggers not being good enough. If I’m good enough then I can prove that I’m not this dirty thing that happened and that it’s not my fault” (Wright et al., 2012, p. 545). This was experienced as a task that was difficult to succeed in by some participants: “Peter told of needing to prove that he was an “OK dad” and reflected that ‘it’s very difficult to prove that you’re innocent” (Dandy et al., 2020, p. 292). This related to, among others, participants having seen how their own parents also had had difficult childhood experiences, thus seeing how abuse was passed down generations. This installed doubt whether they would be able to break the vicious cycle—a doubt that was fuelled by difficulties they experienced in the role as a parent, as explored further in the next theme.

### Theme 2: Own experiences of abuse challenge parents in their new role

Across all studies parents shared how their history of abuse posed challenges to them in their role as parents. Participants from 12 of 13 studies shared how automatic emotional responses tracing back to their own trauma background hindered them in carrying out tasks as parents. In most studies participants also explained how they experienced intense fear, that interfered with emotional closeness to their children and others. Participants in some studies (Byrne et al. 2017; Dandy et al. 2020; Gichaz et al. 2022; O’Brien et al. 2019; Tedgard et al. 2018; Wright et al. 2012) also described more substantial difficulties with being the parent they wanted to be and had little faith in their abilities as parents.

In most studies, participants shared how tasks and situations they encountered as parents triggered memories from their own childhood, often eliciting intense reactions that could be difficult to regulate. Everyday situations, like cleaning their child (Lange et al., 2020), changing diapers (Haiyasoso & Trepal, 2019), and breast feeding (Coles, 2009; Lange et al., 2020) could trigger intrusive memories and strong emotions: “It would cross my mind sometimes with diapering them, just what they looked like—I never touched them or anything like that but just, it would cross my mind as far as that and especially with little boys getting erections” (Wright et al., 2012, p. 544). This could lead parents to avoid situations where they were alone with their child, or where the child was naked, leaving these tasks and situations to their partners, thus feeding their fear of not being good enough parents. But their previous experiences with abuse could also result in a more fundamental lack of trust, that impacted them both as parents and in relation to their partners: “My husband was scrutinized every nappy change which impacted our relationship as a family. However, he accepted this as he knew my history” (Lange et al., 2020, p. 7). Many participants across most studies described how their fear hindered emotional connection with their children. The gender of the child influenced these processes: “I imagine a lot of people who have suffered sexual abuse might think they don’t want a boy, because um, they could possibly be an abuser, although I didn’t want a girl for the reason that I didn’t want her to be abused because I knew I’d love this child and I wouldn’t want any harm to come” (Byrne et al., 2017, p. 472).

Participants in some studies also shared how they encountered significant difficulties as parents, at times failing in their goal of being a good enough parent. For some parents, this related to using their child as support, not being able to be the adult: “I kept her home from school a lot, when she was little, so I wouldn’t have to be alone. You know, she was my best friend in the world really” (Wright et al., 2012, p. 545). Some participants also shared how anger could be triggered in some situations with them failing to regulate this, thus exposing their own children to psychological abuse (shouting, shame induction), or physical violence. These parents shared how they experienced strong feelings of shame and guilt following such situations. But even when parents managed to provide their children with a safe childhood, complex feelings could be triggered: “I envy their childhood. I envy that they have the childhood I wanted without child sexual abuse, without emotional abuse, without physical abuse” (Lange et al., 2020, p. 11). Other parents found it difficult empathizing with their child’s difficulties: “I have trouble relating to my daughter’s problems because I think she’s had such a stable, healthy family life. I think of the troubled childhood I had, and can understand why I had problems with self-image, relationships, and communicating, but feel she doesn’t have a reason to be troubled” (Wright et al., 2012, p. 544).

### Theme 3: Becoming a parent as an opportunity to start a healing process

Across all studies, participants narrated how they experience their whole life changed when they became parents. As seen in theme 2, many participants experienced parenthood as a trigger for strong abuse-related reactions. However, some participants also gained access to a new sense of responsibility and new experiences of being important and valuable for another person through parenthood. In many studies participants also shared how parenthood provided motivation to work with their own issues. For many participants, parenthood thus brought opportunities to seek support and heal old wounds so that they could be the parent they wanted to be.

Many participants across studies shared how becoming a parent provided healing experiences in itself and felt life had taken a positive turn when they had a child: “It was a life changing experience you know having a baby really changes your life, luckily for me it has done for the positive um … it was a journey a good one I wouldn’t change it for the world” (Byrne et al., 2017, p. 472). For some participants, the transition to parenthood provided new meaning and an alternative identity: “It’s the one thing I’ve done right. In 33 years of being on this planet, I don’t ever see that I’ve done much right with my life. (…) You know being a father, creating a life, it is the be all and end all to me” (Dandy et al., 2020, p. 294). For many mothers, experiences with breastfeeding their child also gradually, over time helped normalize the relationship to their own breasts—from a sexualized object to a natural part of their body that filled an important function. Breastfeeding also provided opportunities for experiencing a strong bond with their child, thus a sense of having an important role and a sense of connection: “It’s the love. It’s the giving of my milk to him and sharing with him. I am the only one that can do that for him and it is so strong that love. To have this little baby attached to you makes me feel a really strong connection” (Coles, 2009, p. 319).

The relation they developed with their child and the love they gave and received as a parent was a healing experience in itself for many participants. Many participants shared how they could be very self-critical and experience strong self-hate, but through the love they shared with their child they could heal old wounds and start a process of being more self-compassionate and self-caring: “Yes, I think that having beautiful, open relationships with my own children in which I experience unconditional love for them has helped me to heal a lot of my past experiences” (Lange et al., 2020, p. 11). However, for some participants, experiencing how natural it felt to express love for their own children elicited grief and bitterness towards their own caregivers, who had not provided them with love: “I thought to myself—look at what I lost out on. Suddenly, I was the mother. I was in a new role and I thought—look how much love and how natural it is to give to your child, and to be there for your child” (Kadish, 2015, p. 492). Observing how their own children grew up also stirred up sorrow for what they had missed out on in their own childhood: “She just runs around and plays and has no fears, you know, but I’m there to protect her and when I see that and experience that I miss that part of my childhood. And I’m so glad that she has that, but it makes me sad too” (Wright et al., 2012, pp. 545–546).

In nine of the studies, some participants shared how parenthood also provided motivation to work with their own issues, so they could be better caregivers than the ones they had had: “If I didn’t get help for myself before, just for me, I have to do it now” (Cavanaugh et al., 2015, p. 512). Many participants described how therapy, parent training programs, or self-help books provided support and helped them handle their own reactions and process their experiences of abuse so they could be better caregivers for their children. Several participants underlined the importance of understanding their own automatic responses and how their experiences with abuse impacted them in order to break the cycle of abuse (Matthews & Desjardins, 2019), while other participants experienced an increased fear of repeating the pattern of abuse following increased understanding of how they still were impacted by their experiences of abuse. For some participants, the balance of acknowledging and processing their own reactions and shielding their children from scary experiences therefore became important: “I think that being a survivor you learn to control your feelings so that a lot of times I just keep things hidden, sheltered until I know that I can have the time that I need to deal with it without scaring them or whatever” (Wright et al., 2012, p. 547). Across all studies, participants thus talked about different ways that parenthood provided opportunities for healing experiences.

## Discussion

Our meta-synthesis of how survivors of childhood abuse experience the transition into parenthood showed that the participants in the 13 included studies had a sincere desire to provide their children with a different childhood from what they themselves had lived. They used their own experiences with abuse to navigate what they should not do and to picture a different way of being a parent from what they had experienced. Participants were strongly motivated to succeed in this endeavour. Desiring a good childhood for your child can be viewed as a natural response for a parent that might become even more important for parents with a history of childhood abuse. They do not want their loved ones to have the same experiences as they did.

The participants’ dedication to break the cycle of abuse and their fear of failing in their efforts align with the results both from Herbell and Bloom (2020) meta-synthesis of parenting practices among mothers with a history of abuse and Siverns and Morgan (2019)’s meta-synthesis of parenting in the context of childhood trauma. Our meta-synthesis expands on the existing literature by detailing the desire both mothers and fathers share in providing their children with a better childhood than they themselves experienced, while shedding light on how their experience with abuse makes it challenging to succeed in their endeavour. Our meta-synthesis also expands on Siverns and Morgan (2019)’s descriptions of the healing potential in parenthood by detailing how therapy and external support can be important components in the healing experience.

The participants’ fear of repeating the abuse they had lived, despite their efforts and dedication to break the cycle of abuse, also resonates with the larger literature on trauma and parenting. Research has, for example, shown that the experience of abuse increases the risk of transmitting abuse to the next generation (Greene et al., 2020). However, the same study also found that the majority of parents with experiences with abuse managed to break the cycle of abuse (ibid). Yet, many participants experienced that situations required by parenthood, such as breastfeeding, diapering, or boundary setting, triggered strong abuse-related reactions that created challenges for them. Many participants also experienced that abuse-related interpersonal issues, such as trust, became particularly difficult when sharing responsibility for a child with a partner. Many participants also reflected upon how their own parents had been survivors of abuse, and how abuse could be passed down generations. Across studies we therefore saw examples of participants being torn between a dedication to being a better parent than their own parents, and a fear of passing on the legacy of abuse. This led to many participants doubting their abilities as parents, and many felt shameful for the way they handled difficult situations with their children where abuse-related reactions were triggered.

People who have experienced trauma in their childhood often struggle with guilt, shame and low self-esteem, and often feel torn between their own high standards and their doubt, fear and instinctive reactions. This can lead to demanding spirals of shame (van der Hart et al., 2006). In line with this, many participants in the included studies shared experiences of low self-efficacy in relation to their own abilities as parents, and shame when they experienced failing in their role as a parent. These results also align with previous research that has reported that survivors of childhood abuse often struggle with poor self-esteem and low self-efficacy (Ali et al., 2023; Halvorsen et al., 2020).

This link between a history of abuse and low self-efficacy as a parent is important, as self-efficacy impacts how we experience challenges we encounter (Bandura, 1977). Low self-esteem and self-efficacy can fuel parental insecurity, reflected in the fear many participants expressed for falling short as a parent. This link between parental self-efficacy and the experience of parental challenges might be particularly important to pay attention to in survivors of childhood abuse. Lack of control and being trapped in an unsolvable situation, and disrupted attachment are considered core characteristics of traumatic experiences (Saporta & van der Kolk, 1992, p. 152). Posttraumatic reactions, including strong physiological and emotional reactions being triggered by situations or stimuli that is associated with the original trauma is common among survivors of abuse (van der Kolk, 2014), and might be seen as a continuation of the lack of control constituting the original traumatic experience. Posttraumatic reactions can therefore potentially maintain and aggravate low parental self-efficacy by interfering with necessary parental activities, like breastfeeding and diapering in parents with a history of childhood abuse.

Importantly, across studies participants shared examples of how their dedication to breaking the cycle of abuse along with new experiences provided by parenthood provided opportunities for starting a healing process for their own sake. For some, the relationship with their child provided healing experiences in itself. For others, becoming a parent functioned as a catalyst to seek professional help. An important implication of the results is, therefore, the importance of supporting parents with a history with abuse to strengthen their parental self-efficacy. Potential helpful paths in this direction would be to focus on strengthened regulation skills and self-awareness of how their current reactions are linked to their experience with abuse. This focus is supported by studies that show that therapy can strengthen parental self-efficacy (Maxwell et al., 2016; S. H. Stige et al., 2017). In addition, providing opportunities for positive parental role models seems important. However, given that many survivors of abuse struggle with trust, and many participants fear that child protective services will not see them as fit parents, it is vital to find ways to address these issues that makes it possible for parents to open up about their experiences and seek help. Sensitivity to the potential interference of posttraumatic reactions on parental tasks and a sensitivity to how childhood abuse impacts levels of trust are therefore vital when meeting parents with a history of childhood abuse to lower barriers for help-seeking. This contextual information is also important in understanding the fear many survivors of abuse experience in relation to child protective services taking their children away from them (Blaxland et al., 2022)—thus hindering them in seeking support.

## Methodological reflections

The systematic search constitutes the foundation of a qualitative meta-synthesis. Because the focus of Siverns and Morgan (2019)’s meta-synthesis to a large degree overlaps with ours, it provides unique opportunities to reflect upon challenges in identifying relevant studies to include in qualitative meta-synthesis. Because there only to a small degree are standardized words to identify qualitative studies, searches have to be very broad, resulting in a very time-consuming exclusion process (we went from 4037 potential articles to include 13, while Siverns and Morgan (2019) went from 5288 articles to include 11). In addition to being time-consuming, this situation is associated with a relatively large risk of missing relevant literature, illustrated by the fact that despite having similar searching strategies, eight of the 11 articles included in Siverns and Morgan (2019)’s meta-synthesis was not identified through our search strategy. Similarly, we identified 4 articles published before September 2018 that Siverns and Morgan (2019) did not identify through their search strategy. In addition, we included 6 articles that were published after Siverns and Morgan (2019) did their search. This point to important challenges that the field needs to address to maximize the potential of qualitative meta-synthesis in knowledge generation.

## Limitations

Meta-synthesis represents an important way of aggregating and synthesizing qualitative results within a given topic, thus providing important opportunities to build a more solid knowledge base. There are, however, clear limitations with this method, and with the current article. While representing an important tool to aggregate and present the existing knowledge base within a given field, qualitative meta-synthesis is limited to the research articles that exist within a given field and that are possible to identify through systematic literature searches. The characteristics of the 13 included studies in this article clearly point to limitations in the primary literature, both regarding populations and contexts that have been studied, as well as future directions for research. For example, this meta-synthesis clearly points to how the literature is skewed in relation to gender. Most of the included studies (9 out of 13) focused exclusively on mothers’ experiences of parenthood, and 11 of the 13 included studies only included the perspective of one gender. This means the majority of participants in the included studies are women, but it also means the existing literature to a very small degree can shed light on the interplay between gender and how childhood abuse is experienced to impact parenthood. This has important implications for the transferability of the presented results, but it also points to the importance of exploring fathers’ experiences more in detail and to conducting more studies that include both mothers and fathers in the same study. Given the heterogeneity in focus in the different studies this becomes particularly important.

All included studies were also carried out within one specific context, with no studies explicitly focusing on how culturally informed conceptions of parenthood influence the experience of how a history of trauma exposure impacts the experience of parenthood. Moreover, there is a very scarce literature conducted across quite different cultural contexts. The limited number of studies makes it difficult at present to conduct sub-analysis of how cultural context impacts the phenomena under study. Importantly, we did not identify any studies from low-income countries with less developed welfare systems, identifying to important knowledge gaps. This has important implications for the transferability of the presented findings, but also points to the need for future research to address cultural context more explicitly, and to include participants from a wider range of contexts. Although cultural context clearly will impact conceptualizations and practices of parenthood, as well as availability of support and openness of discussing experiences of abuse, existing literature also supports the existence of common elements across cultures regarding first-person experiences of trauma, trauma reactions, and experiences of attachment between parents and children. Despite obvious limitations, the presented findings still provide an important contribution to the literature by synthesizing existing knowledge across gender and type of abuse.

## Conclusion

In this meta-synthesis, we have explored how survivors of childhood abuse experience the transition into parenthood as both a challenging and a healing experience. We found that all parents expressed a desire to give their children a better childhood, and many felt a fear of not being good enough or continuing the intergenerational cycle of violence. Additionally, several parents struggled with low self-efficacy in their parenting role and had difficulties with emotional regulation in their relationship with their children. Many parents’ fear and shame decreased the likelihood of them seeking help. Therefore, it will be important to further explore how to best support parents with a history of childhood abuse in a helpful way. For many participants, the transition into parenthood was also a source of positive experiences that helped heal relational wounds and increased their confidence in their parenting abilities. This meta-synthesis contributes to the literature by synthesizing both fathers’ and mothers’ perspectives and exploring their experiences beyond a trauma lens. However, the synthesis also points to challenges in identifying qualitative studies for meta-synthesis due to the lack of standardized key words, as well as important knowledge gaps, including the need for more research on fathers’ experiences, the impact on parenthood of a broader range of childhood abuse experiences than CSA. This has important implications for future research.

## Data Availability

All data for this meta-synthesis consist of already published peer-reviewed articles that can be accessed through their respective journals. Articles included as data in this meta-synthesis are marked with * in the reference list.
